# Genetic tests as prophecy: understanding self-defeating and self-fulfilling mechanisms in (predictive) genetic testing

**DOI:** 10.1038/s41431-025-01874-1

**Published:** 2025-06-05

**Authors:** Mayli Mertens, Angus Clarke

**Affiliations:** 1 Center for Ethics, Department of Philosophy, University of Antwerp, Antwerp, Belgium; 2 Center for Medical Science and Technology Studies, Copenhagen University, Copenhagen, Denmark; 3 Atlas Bioethics Center, Andalusia, Spain; 4 Division of Cancer & Genetics, Cardiff University School of Medicine, Institute of Medical Genetics, University Hospital of Wales, Cardiff, UK

**Keywords:** Risk factors, Genetics, Ethics

## Abstract

Predictive genetic tests are reflexive in that they have the potential not merely to *indicate* plausible future health outcomes, but to *influence* the eventual outcome. This article offers an overview of how genetic tests can be self-fulfilling, self-defeating, or otherwise influence what happens to the person’s health. Certain reflexivity is in fact the primary goal of testing, as when those at risk of inherited cancers *intend* to use this knowledge to decrease said risk. Our analysis emphasises unintended, poorly understood, and often overlooked, reflexive effects of predictive genetic testing, as these may become increasingly important in genetic counselling. First, there is reflexivity in predictive testing for Mendelian, ‘monogenic’ disorders. Second, other reflexive mechanisms reveal the potential for feedback loops between genetic susceptibility and expectations held by the self or others—which are even greater, and more complex, in the context of polygenic susceptibility tests for psychiatric illness and cognitive and behavioural traits. Finally, there are additional implications if these tests are used in prenatal genetic testing. These reflexive effects are increasingly likely as genomic testing is more broadly applied to complex diseases and encouraged by trends in personalised medicine—and especially with direct-to-consumer, commercial genetic testing remaining largely unregulated. Recognising the scope of reflexive predictive effects is already useful in genetic consultation and will become more important as the scope of genomic testing broadens to more complex diseases and non-disease traits. Understanding the underlying mechanisms will furthermore increase the possibility of consciously choosing a beneficial response or effective treatment. Without attention to these effects, the consequences of tests for susceptibility to more complex traits are likely to remain opaque and therefore difficult to evaluate and regulate.

## Introduction

How can a predictive genetic test be, appear to be, or become a self-fulfilling or self-defeating prophecy? In other words, how can the result of the test influence what then happens to a person’s health?

Reflexive predictions like self-fulfilling and self-defeating prophecies are predictions that affect the outcome or the way that the outcome is realized. Affecting or controlling health outcomes is the most straightforward purpose of predictive genetic testing for medical or other health purposes. For example, a positive test result is ideally met with a response that will make the prognosis self-defeating. Early detection, or prognostic, or diagnostic tests are as such *intended* to be reflexive [1]. They are intended to give the patient and/or their caregivers some control over how best to respond, before it is too late to do so. However, some of the ways in which the predictions are performative are unintended and poorly understood, especially when some of the responses to the prediction happen subconsciously in the mind and body of the person in question. With this article, we offer a first overview of the different ways in which (predictive) genetic information can impact health and disease progression, identifying which of those are easy to understand and where our understanding is currently lacking.

## Methods

Our approach has been reflective and analytical, not experimental. We have drawn on clinical experience and examples of relevant literature, including experimental studies, to analyze reflexivity in predictive and susceptibility genetic testing. To do this, we used a theoretical framework for analyzing self-fulfilling prophecy in medicine [1] and other practical and automated predictions [2]. The framework was initially developed in the context of neuroprognostication after hypoxia related to cardiac arrest [3, 4].

In short, self-fulfilling reflexivity requires four conditions: credibility, employment, employment sensitivity, and self-fulfillment. The first three conditions are sufficient for any reflexivity, ranging from self-fulfilling to self-defeating reflexivity. For clarity, we illustrate the conditions by means of a straightforward example: self-defeating reflexivity in genetic testing for *BRCA1*. For a positive test result to become self-defeating, the person at risk of breast cancer must first give sufficient *credibility* to the test result to act upon it. If this first condition is met, a response might follow. For example, the decision to get a double mastectomy is an *employment* of the prediction; the second condition. Employment sensitivity is the third and most complicated condition. Basically, it means that something in the system that the prediction is about (typically, in medicine, the health of the patient) is sensitive to the prediction and its employment. Because the reflexivity in the case of *BRCA1* is intended, we understand the *employment sensitivity* in this case well: the potential development of breast cancer is sensitive to whether or not there is breast tissue in which it might develop. Hence, removing the breast tissue is an employment which the system is sensitive to. In this case, the 3 conditions result in self-defeating reflexivity as the fourth condition. Importantly, this *reflexivity* can be operative or transformative. In both cases it is the prediction and its employment that created the outcome. However, *operative reflexivity* doesn’t change the outcome, only how it was brought about. For instance, when a person doesn’t develop breast cancer because of the double mastectomy but wouldn’t have developed breast cancer anyway (false positive). *Transformative reflexivity* changes the outcome itself from what it would have been had no prediction taken place e.g. the person does not develop breast cancer but would have developed it had no double mastectomy been performed (true positive). The operative-transformative distinction has epistemic and moral implications which are discussed elsewhere [1–4]. A test may also be self-fulfilling, meeting the fourth condition of a reflexive effect that leads to self-fulfillment, as can happen when a diagnostic genetic test identifies a variant often associated with a poor neurological outcome. In the context of whole genome sequencing (WGS) applied to sick infants in a neonatal or pediatric intensive care unit, a poor prognosis may lead to the withdrawal of intensive care and a switch to palliative care or to sedation and hydration only. This has the potential to become a self-fulfilling prophecy, for example if the infant is dependent on a mechanical ventilator for respiration. If the child would have died in any case then this effect is operative; if the prognosis was unduly bleak, so that the child could in fact have survived in fair health, then the effect will have been transformative: it will have altered the outcome for the worse. This is comparable to the setting in which our theoretical framework was developed – i.e. the neuroprognostication performed on victims of cardiac arrest being ventilated on intensive care units.

In this article, we identify and include reflexive mechanisms that may not be intended and, hence, may be unforeseen and overlooked. We offer a first overview of the reflexive landscape in genetics, insofar as the employment sensitivities can be scientifically explained. Towards the end, we suggest potential pathways for future research that could explain some employment sensitivities that are currently beyond our understanding, and for which the theoretical framework can help the analysis. The framework was previously used for analyzing self-fulfilling reflexivity in neuroprognostication and intensive care [3, 4], emergency medicine [5], fertility treatment outcomes [6], the use of AI in medicine [1–5], and other practical and automated types of prediction in education, crime, credit scoring, etc [2]. The case of genetic testing helps to further broaden the framework to other types of reflexive prediction and make it more robust. The results were achieved through application of different cases to the model, critical analysis and reflection, and discussion of our intermediate findings with other experts in (epi)genetics.

## Results

### Monogenic conditions

First, and most obviously, an unfavourable (‘positive’) result may be self-defeating if the patient is able to use the test result to access medical care that influences their health for the better. In the hereditary cancers of the breast and bowel and some of the less common familial cancer syndromes (e.g. Multiple Endocrine Neoplasia syndromes and von Hippel-Lindau syndrome), knowing you are at risk can trigger entry to a tumour surveillance programme and/or lead to surgical interventions to remove high risk organs, as was the case in the breast cancer example discussed above. This is what the genetic test is designed to enable. From a near certainty of death from bowel cancer, someone with familial adenomatous polyposis coli (FAP) can have a colectomy performed and the risk then becomes greatly reduced. Similarly, someone at high risk of coronary artery disease from familial hypercholesterolaemia can take statins to reduce their blood cholesterol and thereby their risk of myocardial infarction. Ensuring that the positive test result is self-defeating is therefore the goal for conditions that have known effective treatments.

The context of many neurodegenerative disorders, where there are no established pre-symptomatic medical interventions, is somewhat different. There will be real benefits for some patients in terms of life planning, such as the reproductive decisions being made by the individual or others in their family with whom they share the test results. Even where there is (so far) no direct therapy to offer, there can be broader benefits to the patient and/or their family. In such cases, when the test does not affect the health progression of the patient in an intended, straightforward way, the genetic test will neither be fully self-fulfilling nor self-defeating but it can nevertheless be reflexive in varying degrees. The reflexivity can be operative or transformative [1–4] by impacting the patient’s context, their psychological wellbeing, or the way in which they cope with the possibility of disease—all of which can *operatively* impact the timing of the disease’s progression and *transformatively* impact the severity of the disease. An example of transformative reflexivity is when someone predicted to be at higher risk of some kind of cognitive decline starts doing a lot of cognitive training. While the onset of the disease may still occur at the same time, the symptoms may be less debilitating because a larger buffer has been built between the cognitive erosion and the basic cognitive functions that allow independent living. When the same debilitating symptoms occur but at a later stage in time, this is an example of operative reflexivity.

Sadly, both forms of reflexivity work in the opposite way too. For example, when stress, anxiety, or worry, speed up cognitive erosion, in time and/or degree. A study on the *APOE*-genotype, a genotype associated with increased risk of Alzheimer’s disease, showed that merely informing healthy older adults of their *APOE* genotype affects their subjective memory performance and, in cases of pessimistic information, it affects even their objective memory performance to match the information received [7]. As such, an adverse (positive) predictive genetic test may unintentionally be self-fulfilling. Unfortunately, the potential for such effects is often ignored in reviews of *APOE* testing and cognition, as it is in an otherwise most interesting review of the effects of *APOE* status on cognition in midlife, which attends to other environmental circumstances and potential interactions [8].

An adverse predictive result may also *appear to* trigger the onset of a Mendelian neuro-degenerative disorder like Huntington’s disease if the patient seeks testing because of subtle, early features of the disease, such as clumsiness or minor lapses of memory. As before, this effect arises when the test is for a genetic factor of major effect and the condition is essentially Mendelian. The at-risk patient may be working hard, struggling perhaps, to minimise the overt symptoms that others would notice. With an adverse result, not only will time have passed leading naturally to a worsening of symptoms during the process of ‘counselling and testing’, but the patient may become depressed or simply choose to cease efforts to minimise the overt features of the disorder. Indeed, this loss of motivation to resist the symptoms may itself be a feature of the condition. Family members observing this can become convinced that the test result itself led to the onset of the disease. The test may not always be *substantively* self-fulfilling in that the physiological progression of the condition is affected, as when the patient’s response to the test worsens the expression of the disease. The patient’s and/or their family’s and friends’ perception of the symptoms’ progression may change to nevertheless cause *interpretative* self-fulfilment, interpreting the outcome more negatively than they would have, had there been no testing [1–4]. Both forms of unintended reflexivity may be prevented by educating the patient about the possibility of such undesirable effects and, with or without additional psychological support, making them more resilient to unfavourable news.

Finally, a patient may inappropriately interpret a negative test result (“good news”, when the family’s known pathogenic variant is shown to be absent) as indicating an absence of risk in either of two scenarios. These can then be self-defeating in a harmful sense. One situation arises when a laboratory or other error mistakenly indicates a lack of risk. While unusual, false negative test results are likely to be self-defeating in that it takes away the patient’s ability to seek precisely the medical care that they could get to prevent the development of the condition. In the Multiple Endocrine Neoplasia syndromes and von Hippel-Lindau syndrome, for example, the falsely favourable result may reassure the patient so that they do not join the tumour surveillance programme and their condition may worsen to the point that surgical intervention comes too late. More common than false negative test results are misapprehensions that a negative *BRCA1* or *BRCA2* test result in a woman at risk of familial breast and ovarian cancer means the patient will not develop a breast cancer. In fact, of course, her risk is reduced to no less than the still substantial population lifetime risk of some 10% [9]. This misapprehension could lead a woman to ignore early signs of a breast tumour, so that the prediction of reduced risk could thereby become (unfortunately) self-defeating.

### Complex disease and susceptibility tests

The reflexive aspects of predictive genetic testing described above are surely to be expected as elements of the context within which predictive tests are situated. Furthermore, these elements do not undermine the basis on which the tests are performed but arise from it, as natural human responses to the possibility of such predictive knowledge. The context will be very different, one may imagine, if tests for susceptibility to more complex traits are made available, as is beginning to happen through commercial channels of more or less propriety that apply the concept of the polygenic score for a complex trait or disease. These tests are currently less likely to be offered within healthcare, as the utility of polygenic tests for complex traits – such as behaviours, abilities, and degrees of susceptibility to specific diseases – is much less than for strongly predictive tests for clear diagnostic entities [10]. Indeed, recent authoritative reviews of the clinical applicability of polygenic scores establish that their application would be at best premature. Lacking evidence of utility and consistency [11, 12], their application may be shown to be useful in future but largely restricted to specific purposes in population health programmes [13]. In contrast to the situation of high penetrance genetic conditions, medical interventions aimed at modest differences in susceptibility to disease are much less likely to have a substantial impact. Furthermore, the prospect of other benefits from test results is much less. Thus, we have no good reason to expect helpful responses in terms of behaviour change [14, 15] or psycho-emotional equilibrium, so that the possibility of health outcomes being distorted adversely by the results themselves is much greater [16]. Reports suggesting that polygenic score results indicating an increased risk of disease lead to ‘medically appropriate’ behaviour change are mostly rather optimistic interpretations of short-term studies that take at face value statements made by research project participants that they have made the appropriate behaviour changes but without evidence that these statements are accurate and without any evidence of subsequent health outcomes. Thus, in one study, at 18 months after a clinical and genomic risk assessment, 42.6% of those at >10% risk of a cardiovascular event - as against 33.5% of those at lower or average risk - said they had made some appropriate behavioural change. The odds ratio for self-reports of having made a favourable behavioural change in response to the polygenic score results was 1.1 (confidence interval 1.03–1.17), but with no information about health outcomes (Widen et al.) [17]. We interpret this as providing weak evidence of good intentions sustained for 18 months but no evidence of a beneficial impact on outcomes.

This scepticism about the beneficial effects of reported behaviour change is not to deny that such scores could in the future be helpful in a different context, that of stratifying disease risks in population screening programmes, but a substantial amount of work will be required to integrate these results into the appropriate clinical pathways [10]; they are much more likely to be useful in the implementation of health screening than in making prognostic decisions about individual patients and, therefore, it will not be possible to determine the accuracy of these prognoses (these ‘predictions’ of disease risk).

There are at least three mechanisms through which such tests of susceptibility may lead to (often adverse) distortions of health outcomes. Two of these operate directly through the patient’s belief that they will or will not be affected by the condition tested for, while the third category operates less directly but through the emotional turmoil triggered by an adverse result.

One direct effect of being given a (mildly) favourable risk result could be the misapprehension that one is no longer at risk of disease, so that the usual ‘healthy lifestyle’ advice that applies to all of us can safely be ignored [16]. In other words, the patient feels *invulnerable* so that healthy diet and exercise advice, for instance, can be ignored. To the extent that this happens, the individual’s risk of disease will increase, as disease susceptibilities operate in a whirl of competing influences on disease risk. The genetic factors tested for are likely to play only a modest part in this, alongside environmental influences, life-history events mediated through epigenetic effects, and simple chance. This is a test result that undermines itself in its effects. To the degree it is effective in doing so, it is self-defeating. In contrast, the same invulnerable feeling may trigger a placebo effect that somehow ensures self-fulfilling reflexivity, even in cases where the (mildly) negative test result may have been false. In scenarios where both mechanisms are at play, there will be feedback loops between the two that further complicate analysis of reflexivity on the outcome.

The other direct effect is a self-fulfilling prophecy that operates through the sense of *fatalism* that may apply if someone is told that they are at a (mildly) increased risk of disease: they may feel they will develop the condition however they behave, so they see no point in attending to the relevant lifestyle factors, such as diet and exercise in the case of many of the complex degenerative disorders prevalent in developed societies. Their behaviour may then contribute to the disease that they could have worked harder to avoid. This is not to say that the person is necessarily responsible for their health outcome, as other socio-economic factors may make it a lot harder for some individuals to access better lifestyle options [18]. In such cases, there is definitely a societal component to be considered in the self-fulfilment of the test result. Even if no undermining actions are undertaken, simply believing that disease is inevitable may cause a nocebo effect. If both are at play, they are likely to reinforce and amplify the effect. As such, even if the positive test result is false, the response may cause *transformative* self-fulfilment, effectively changing the outcome from what it would otherwise have been.

In addition, and just as for the strong, Mendelian genetic factors, this same test result of being at (mildly) increased risk of disease may become happily self-defeating if it sparks a *valiant, defiant* or *(medically) virtuous* attitude in the person, who will change their lifestyle significantly based on the prediction. While this may be difficult as a sustained response, it would still be a constructive response that might achieve benefits for the individual. In fact, the emerging cultures focused on self-tracking, self-improvement, and enhancement show that this kind of reflexivity is likely to become more common [19]. However, here too, whether healthier options are readily available and whether the socio-economical context invites or supports such a valiant attitude is also an important contributing factor to whether the test result can become self-defeating.

The possibly more complex reflexive effects of susceptibility test results may apply in relation to mental health, behaviour, and cognition. If someone is at a somewhat increased risk of developing schizophrenia, and given that the chance of this developing can be influenced by the individual’s emotional equilibrium and the level of expressed emotion in their environment, there are several potential links between the test result and the onset of a disease episode. The individual’s own emotional state may be disturbed by the result and, furthermore, their emotional environment may be more challenging if their family, or others who know them, are aware of their test result. In contrast, one may imagine a scenario where the increased risk signals to the individual and their family an opportunity to learn more about the condition and possible prevention strategies. The increased support and attention from their environment may boost the individual’s emotional equilibrium and confidence in such a way that the onset of a disease episode is avoided or mitigated. These interactions between test result and disease causation are different and certainly more complex than the relatively simple cases of invulnerability, fatalism, and even valour considered above in relation to recommendations about diet and exercise. However, the risk of self-fulfilling and self-defeating reflexivity and their potential looping effects still apply and might be relevant in other mental health domains, such as the risk of depression and mania.

In this area, the scope for potential effects is very substantial but poorly understood. To illustrate, in families where a chromosomal copy number variant (CNV) segregates that is recognised as a neurosusceptibility locus, there is a potential for family members to inspect each other (and each other’s behaviours) to see who might also carry (and be somewhat affected by) the family’s CNV. We have seen this effect operate within families. Family members may then attribute any quirk or perceived personality flaw to the CNV. Such attributions could of course be seen as either exonerating family members for their idiosyncrasies or as blaming them for the same characteristics, engaging the family’s attention, whether or not the individuals so assessed are ever tested for the CNV. While this interpretative reflexivity may be unsupported by scientific evidence, it can have some substantive effect on the mental or physical health of the individual in question, especially when they themselves adopt this (potentially false) belief as true.

Furthermore, beyond questions of mental health, these considerations can be applied to the already challenging case of social and behavioural genomics [20] and other non-disease complex traits such as cognition, most prominently the IQ score. When an IQ test gives a result below the mean, although within the normal range, there is a real possibility that a lack of confidence on the part of the individual could influence their educational attainment. Even more damaging, perhaps, could be knowledge of the test result if it is available to those in authority. If a classroom teacher holds low expectations of a child’s performance, this is known to influence educational attainment in self-fulfilling ways [21, 22]. In the same way, polygenic tests that predict a likely range for a person’s IQ score could exert similar influences, helping or hurting the attainment of a child and becoming self-fulfilling prophecies, in either substantive or interpretative ways [2]. Similar considerations may be seen to apply to challenging behaviours, such as the predisposition to violence, even if there are cases where the border between such non-disease behavioural traits and a disease state will be blurred, as when attempting to determine whether an episode of violence is the result of a mental health problem (paranoia, for example).

### Prenatal genetic testing

A final category of self-fulfilling prophecy to consider is the application of genetic tests to prenatal screening programmes or the related context of preimplantation genetic diagnosis. Imagine that a nation’s health services offer a screening test to every pregnant woman that enables her to avoid giving birth to children with a genetic condition such as Down syndrome or another genetic cause of disability. Such a policy is known to lead to societal consequences that impact on those affected individuals who are born, on the parents who decide not to take part in such screening and then have affected children, and on those who find that they have an affected foetus but at that point determine, possibly against the expectation of others and even against their own provisional ‘decision-in-advance’, to continue the pregnancy [17].

Several studies have shown that parents do regularly make different decisions in advance from how they decide to act when confronted with the real prospect of terminating their pregnancy [23, 24], and that this may relate to when (at what stage in pregnancy) they find themselves to be committed to their pregnancy [25]. These unplanned decisions may then lead to increased stigmatisation of affected individuals (and their parents). There may be financial penalties, in terms of reduced societal provision of support to meet special health care and educational need [26], that could be classed as social discrimination. Importantly, such impacts would be self-fulfilling interpretatively in the former case, and substantively in the latter, causing feedback loops that further confirm and amplify the notion that these conditions are indeed creating a poor quality of life that should be avoided [4]. This is especially true for cases where a reduced quality of life is felt mostly through the negative societal response to the condition, or through the lack of societal support [1].

Especially concerning are prenatally diagnosed variants of uncertain significance that “leave families with complex and unclear information they cannot act upon with confidence” [27]. Generally, the more uncertain the information, the more likely that the actions that follow it will be reflexive in unintended ways. Furthermore, actions that follow predictive information are intended to control and protect against risk, thereby often restricting the range of potential outcomes. Uncertain information has a higher likelihood of being false, in which case the restrictive action is more likely to be just that—restrictive.

## Discussion

In conclusion, there are clearly multiple possible routes through which a genetic test result can influence the outcomes for those tested and some of these deserve to be regarded as self-fulfilling or self-defeating prophecies. Crucially, reflexive genetic tests become self-defeating or self-fulfilling depending on the response to (or employment of) the test result. While some of those responses are very conscious, explicit, and purposely intended to be reflexive, many responses are un- or subconscious, implicit, and often poorly understood. Because we understand the reflexive mechanisms in conscious, explicit responses, it is easier to exert control over how predictions can intentionally impact the health outcome. However, it is a lot harder to gain control over the un- or subconscious, implicit, and often complex responses of individuals and others in their medical and social networks.

With increased understanding, such intangible mechanisms could be put to therapeutic use. In this way, their unintended reflexivity could be turned to intended reflexivity. And, as some have argued, non-harmful strategies that can be used to foster or improve health, ought to be utilized [28]. For this reason alone, currently elusive responses to (predictive) genetic information require further analysis. Well-known but still poorly understood examples of such implicit responses are placebo and nocebo effects. Historically, these have been put to therapeutic use even when they were not well understood [29]. In modern times, there has been renewed interest in the power and effectiveness of these effects. Even mock surgeries have recently been restudied and found to be equally successful as the real surgical interventions [30]. Moreover, to respect patient autonomy and to avoid malpractice based on misinformation and trickery, studies have been done to measure placebo effect *while* transparently informing the recipients of the administration of placebo [31]. Surprisingly, the curative effects remained positive, motivating physicians to recommend placebos when they are open-label [32]. Such interventions could go as far as explicitly emphasising the lack of pharmaceutical or physiological impact as well as the intended effect, including the potential for reflexive impact.

Practical impact for prognosis, sharing of risk information, and possible intervention remains limited by outdated understanding. Mapping in what ways risk information and subsequent responses can be reflexive, and therefore looking for employment sensitivity, is a great way to start but practitioners can only implement the knowledge they possess. The solution to this limitation is more research into different forms of plausible employment sensitivity, especially of the kind that ventures away from old assumptions in genetics. Fortunately, the mechanisms behind placebo, nocebo, and other relations between mind or mindset and physiology, are increasingly becoming apparent through intensive study, including effects of non-pharmaceutical impacts on brain circuitry, neurotransmitters, hormones, and the gut [33–37]. Perhaps the currently most impenetrable impacts of genetic predictions are those that have an impact on gene expression or genetic risk itself [38] and the feedback loops between genetics and prediction due to phenomena like the Placebome, i.e. genetic information that impacts the placebo response [39]. New insights emerging from Michael Levin’s multidisciplinary work combining ideas in cognitive science, evolutionary biology, and developmental physiology [40] may be worthy of further exploration. To untangle the currently most mysterious forms of employment sensitivity, future research may, for example, investigate the connection between cognition and bioelectric changes.

Insofar as insight is gained, the understanding of reflexive mechanisms and the possibility of consciously choosing a beneficial response or effective treatment will increase too. It may also influence the decision on whether or not to receive test results. As long as such insight remains obscure, however, and especially when no established pre-symptomatic medical interventions are available, we argue that tests for susceptibility to more complex traits are likely to cause more harm than good.
